# An NB-IoT-Based Architecture with Spatial-Statistical Analytics for Cross-Domain Air and Water Quality Monitoring in Aquaculture and Aquatic Environments

**DOI:** 10.3390/s26185964

**Published:** 2026-09-20

**Authors:** Tsai-Chen Yang, Yin-Tzu Huang, Yu-Fang Chung, Tzer-Shyong Chen, Chao-Tung Yang

**Affiliations:** 1 Department of Computer Science, Tunghai University, Taichung City 407224, Taiwan; cynthiayang1106@gmail.com; 2 Global Master of Business Administration, Tunghai University, Taichung City 407224, Taiwan; deannehuang@thu.edu.tw; 3 Department of Electrical Engineering, Tunghai University, Taichung City 407224, Taiwan; yfchung@thu.edu.tw; 4 Department of Information Management, Tunghai University, Taichung City 407224, Taiwan; 5 Research Center for Smart Sustainable Circular Economy, Tunghai University, No. 1727, Sec. 4, Taiwan Boulevard, Taichung City 407224, Taiwan; 6 Department of Medical Research, Kuang Tien General Hospital, Taichung City 43304, Taiwan

**Keywords:** Narrowband IoT (NB-IoT), precision aquaculture, cross-domain correlation, air quality monitoring, water quality monitoring, environmental sensing, dissolved oxygen, partial correlation, temporal autocorrelation

## Abstract

The rapid expansion of smart agriculture and precision aquaculture necessitates continuous, high-resolution environmental monitoring to optimize ecosystem stability and prevent catastrophic biomass loss. Conventional Internet of Things (IoT) solutions routinely treat atmospheric and aquatic parameters as isolated domains, neglecting the dynamic physicochemical coupling occurring across the air–water boundary layer. To overcome this domain fragmentation, this study presents an integrated edge-cloud telemetry architecture designed for concurrent, multi-domain environmental monitoring and cross-domain spatial-statistical analysis. The proposed framework employs low-cost, multi-sensor edge nodes integrated with Narrowband IoT (NB-IoT) cellular communication, achieving high signal penetration, energy-efficient operation, and direct base-station connectivity without local gateway dependencies. The system continuously acquires atmospheric parameters (temperature, relative humidity, particulate matter ${\mathrm{PM}}_{1.0}$/${\mathrm{PM}}_{2.5}$/${\mathrm{PM}}_{10}$, ozone $O_{3}$, total volatile organic compounds TVOC, equivalent ${\mathrm{CO}}_{2}$, Air Quality Index AQI, and Ultraviolet Index UVI) alongside aquatic indicators (water temperature, pH, dissolved oxygen DO, electrical conductivity EC, and turbidity). Telemetry is streamed via Message Queuing Telemetry Transport (MQTT) to a centralized MySQL cloud database, providing real-time Grafana dashboards, spatial Inverse Distance Weighting (IDW) mapping, and automated multi-channel alerting via LINE Notify and email. The architecture was deployed and validated across the Tunghai University aquatic research facility, capturing *n* = 14,400 synchronized 1-min observations (with an initial raw Packet Delivery Rate of 99.24%). Statistical evaluations accounting for temporal autocorrelation ($N_{\mathrm{eff}}\approx 892$) and False Discovery Rate correction revealed significant cross-domain associations ($p_{\mathrm{adj}}<0.001$), notably an inverse association (r=−0.782, ρ=−0.794, τ=−0.612) between ambient air temperature and aquatic dissolved oxygen physically consistent with Henry’s Law of gas solubility, an inverse association (r=−0.763) between atmospheric humidity and dissolved oxygen, and a positive association (r=+0.789, ρ=+0.812) between humidity and aquatic turbidity. First-order partial correlation analysis ($r_{T_{a},\mathrm{DO}|T_{w}}=-0.172$) confirmed that water temperature serves as the primary thermal mediator of dissolved oxygen depletion. By synergizing low-power NB-IoT telemetry with robust multi-domain analytics, this work provides a scalable, empirical foundation for transitioning from reactive threshold alerting to proactive predictive management in precision aquaculture.

## 1. Introduction

As global food security challenges intensify, aquaculture has emerged as a cornerstone for delivering sustainable, high-protein nutrition worldwide. Modern aquaculture and aquatic farming practices are, however, acutely vulnerable to environmental volatility. Fluctuations in water quality—such as sudden depletion of dissolved oxygen (DO), extreme pH shifts, or sudden spikes in electrical conductivity and turbidity—frequently lead to severe physiological stress, increased disease susceptibility, and catastrophic biomass mortality [1,2,3]. Consequently, the transition from manual, sporadic water sampling to automated, real-time smart aquaculture monitoring is vital for the modern blue economy [4,5].

The rapid adoption of the Internet of Things (IoT) has catalyzed significant advances in precision environmental monitoring [6,7]. Nevertheless, the vast majority of existing monitoring systems remain fundamentally domain-isolated. Conventional platforms monitor either atmospheric variables (e.g., ambient temperature, relative humidity, particulate matter concentrations) in terrestrial/greenhouse settings [8,9,10] or aquatic physicochemical parameters (e.g., pH, DO, temperature, conductivity) in aquaculture ponds [5,11]. This siloed paradigm fails to account for the continuous thermodynamic, chemical, and biological fluxes that govern the atmospheric-aquatic boundary layer. For instance, ambient solar radiation, atmospheric thermal retention, boundary-layer humidity, and aerosolized particulate deposition correlate with water column temperature equilibration, gas solubility, surface evaporation, and microbial/algal proliferation [12,13].

To address this crucial gap, this study proposes an integrated telemetry and spatial-statistical monitoring architecture specifically engineered for synchronized, cross-domain atmospheric and aquatic environmental monitoring. The system leverages an array of low-cost, multi-parameter sensor nodes coupled with Narrowband IoT (NB-IoT) cellular communication [14,15]. NB-IoT was deliberately selected as the core communication backbone due to its licensed spectrum reliability, superior indoor and structural penetration capability in humid aquatic environments, minimal power consumption supporting extended battery autonomy, and direct cellular base-station connectivity that eliminates the requirement for deploying and maintaining dedicated local gateways.

The primary objective and core contribution of this work is the rigorous empirical quantification of cross-domain relationships linking atmospheric conditions with aquatic responses. Telemetry streams from distributed edge nodes are routed via the Message Queuing Telemetry Transport (MQTT) protocol to a centralized cloud architecture hosted on MySQL and Grafana [16,17], enabling real-time telemetry visualization, automated threshold-based multi-channel notifications (LINE Notify and Email), spatial Inverse Distance Weighting (IDW) interpolation mapping, and multi-metric statistical correlation analyses (Pearson, Spearman, and Kendall) alongside mechanistic thermodynamic evaluations.

The principal contributions of this paper are summarized as follows:

1. **End-to-End Cross-Domain Monitoring Architecture:** We design, implement, and field-deploy a scalable, multi-tier architecture integrating low-cost sensing nodes, NB-IoT cellular telemetry, secure MQTT brokering, relational persistence, and interactive visualization for simultaneous atmospheric and aquatic monitoring without on-site gateway dependencies.
2. **Empirical Cross-Domain Correlation and Confounding Analysis:** We provide a comprehensive statistical evaluation (Pearson *r*, Spearman ρ, Kendall τ, moving block bootstrap 95% confidence intervals, effective sample size $N_{\mathrm{eff}}$ autocorrelation adjustment, and FDR multiple testing control) quantifying critical inter-domain interactions. Furthermore, partial correlation analysis explicitly isolates the mediating role of water temperature on dissolved oxygen depletion.
3. **Practical Validation and Metrological Framework:** We validate the system across real-world aquatic research facilities at Tunghai University, establishing complete calibration transfer models with empirical $R^{2}$ and RMSE metrics, communication reliability metrics (Packet Delivery Rate 99.24%, latency 1.82±0.34s), data imputation reconciliation, and baseline guidelines for proactive aquaculture environmental control.

The remainder of this paper is structured as follows: Section 2 reviews related literature in smart aquaculture and environmental IoT. Section 3 details the system architecture, hardware nodes, calibration protocols, and cloud software infrastructure. Section 4 presents the experimental deployment, spatial mapping, and cross-domain empirical correlation results. Section 5 discusses the thermodynamic and biophysical mechanisms underpinning our findings alongside operational implications. Finally, Section 6 concludes the paper and outlines future directions.

## 2. Related Work

### 2.1. IoT Systems in Environmental and Aquaculture Monitoring

Recent advancements in IoT sensing and telemetry have transformed environmental telemetry from periodic manual grab-sampling into automated continuous telemetry networks [4,6]. In atmospheric monitoring, extensive efforts have focused on quantifying urban air pollution and greenhouse microclimates using low-cost electrochemical and optical particle counters [8,18,19]. For example, Chen et al. [9] and Lin et al. [10] implemented distributed sensor grids to track fine particulate matter (${\mathrm{PM}}_{2.5}$) and volatile organic compounds across industrial and agricultural zones.

In parallel, aquaculture telemetry systems have concentrated on monitoring core water parameters essential for aquatic health, including pH, water temperature, dissolved oxygen (DO), and electrical conductivity [5,11]. Sampaio et al. [2] emphasized that unmonitored dissolved oxygen deficits induce acute hypoxia in cultured fish, precipitating catastrophic mortality within hours. To address this, various microcontroller systems based on Arduino, ESP32, and Raspberry Pi platforms have been deployed in freshwater and marine farms [20].

### 2.2. Communication Protocols for Remote Aquaculture Sensing

Telemetry in aquatic and agricultural environments presents severe propagation challenges, including high humidity, water surface RF reflections, and physical obstructions [14]. Traditional short-range wireless technologies such as ZigBee, Bluetooth, and Wi-Fi suffer from severe transmission range limitations, high power consumption, and poor penetration through reinforced concrete pond structures [15,21].

Consequently, Low-Power Wide-Area Networks (LPWAN) have gained widespread prominence [14]. While LoRa and LoRaWAN offer long-range, license-free transmission, they necessitate the deployment, maintenance, and powering of dedicated outdoor gateway hardware, which introduces a single point of failure in remote or distributed farming sites [15]. In contrast, Narrowband IoT (NB-IoT), standardized by 3GPP in Release 13, operates within licensed cellular spectrum bands. NB-IoT provides an enhanced maximum coupling loss (MCL) of 164 dB (yielding +20 dB link budget gain over conventional GPRS), exceptional structural penetration, carrier-grade quality of service (QoS), and direct connectivity to established cellular base stations [15,22]. These attributes make NB-IoT uniquely suited for robust aquaculture deployments where gateway installation is logistically unfeasible.

### 2.3. Cross-Domain Environmental Modeling and Research Gaps

Despite substantial progress in single-domain sensing, existing platforms almost exclusively isolate air quality from water quality. Table 1 provides a comprehensive comparative synthesis benchmarking the proposed system against representative state-of-the-art environmental monitoring platforms.

As delineated in Table 1, existing platforms remain domain-segregated, lacking concurrent cross-domain telemetry and multi-metric statistical correlation frameworks. By deploying synchronized multi-channel sensing coupled with rigorous non-linear, non-parametric, and partial correlation models, this work bridges the critical divide between atmospheric conditions and aquatic biophysical dynamics.

## 3. System Architecture and Implementation

### 3.1. Overall System Architecture

**Figure 1:**
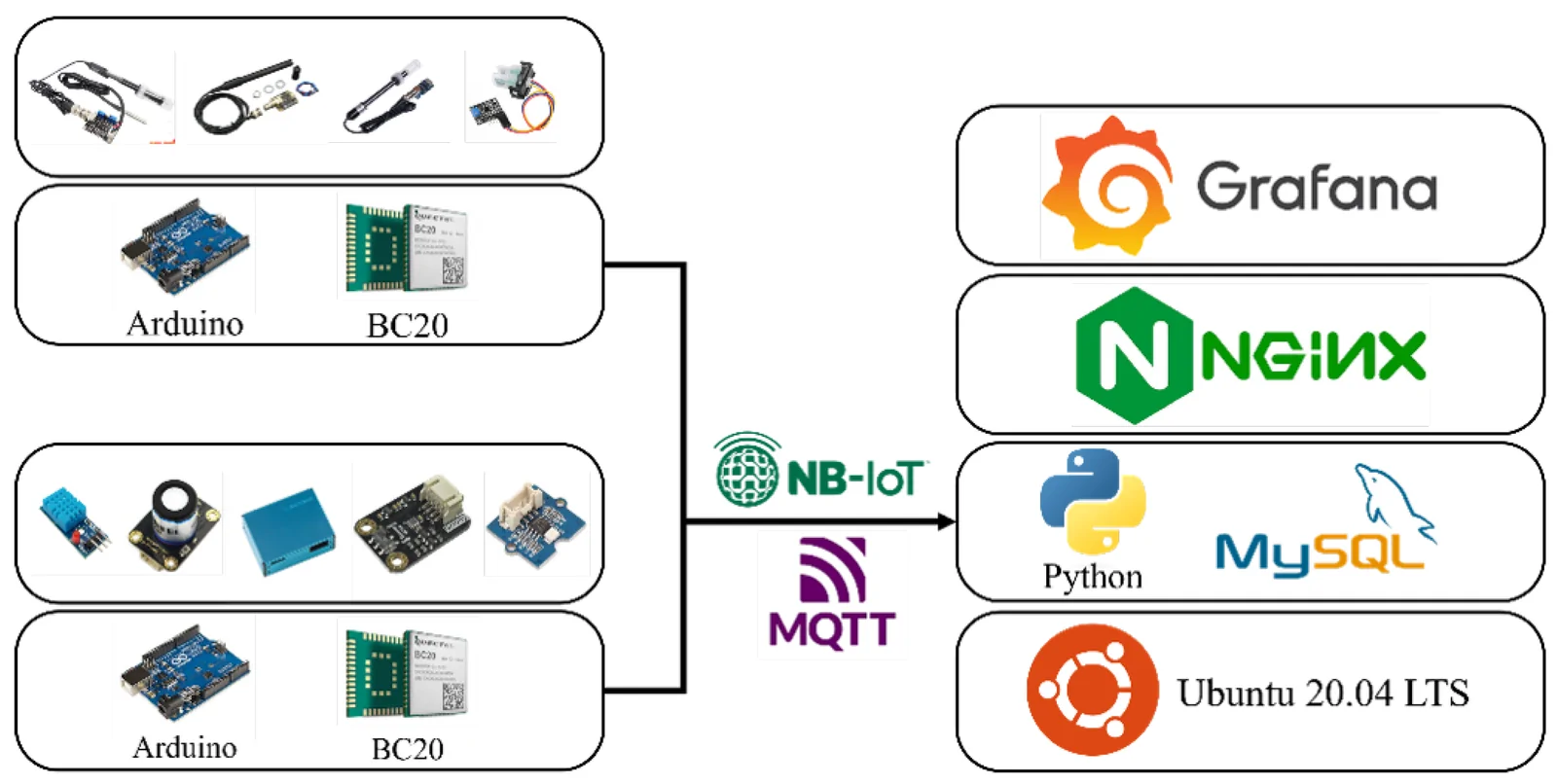
Four-tier architecture of the proposed cross-domain monitoring platform.

The overall architecture of the proposed cross-domain monitoring platform is structured into four functional tiers: Edge Sensing Layer, Telemetry and Network Layer, Cloud Ingestion and Persistence Layer, and Application and Visualization Layer, as illustrated in Figure 1.

**Figure 2:**
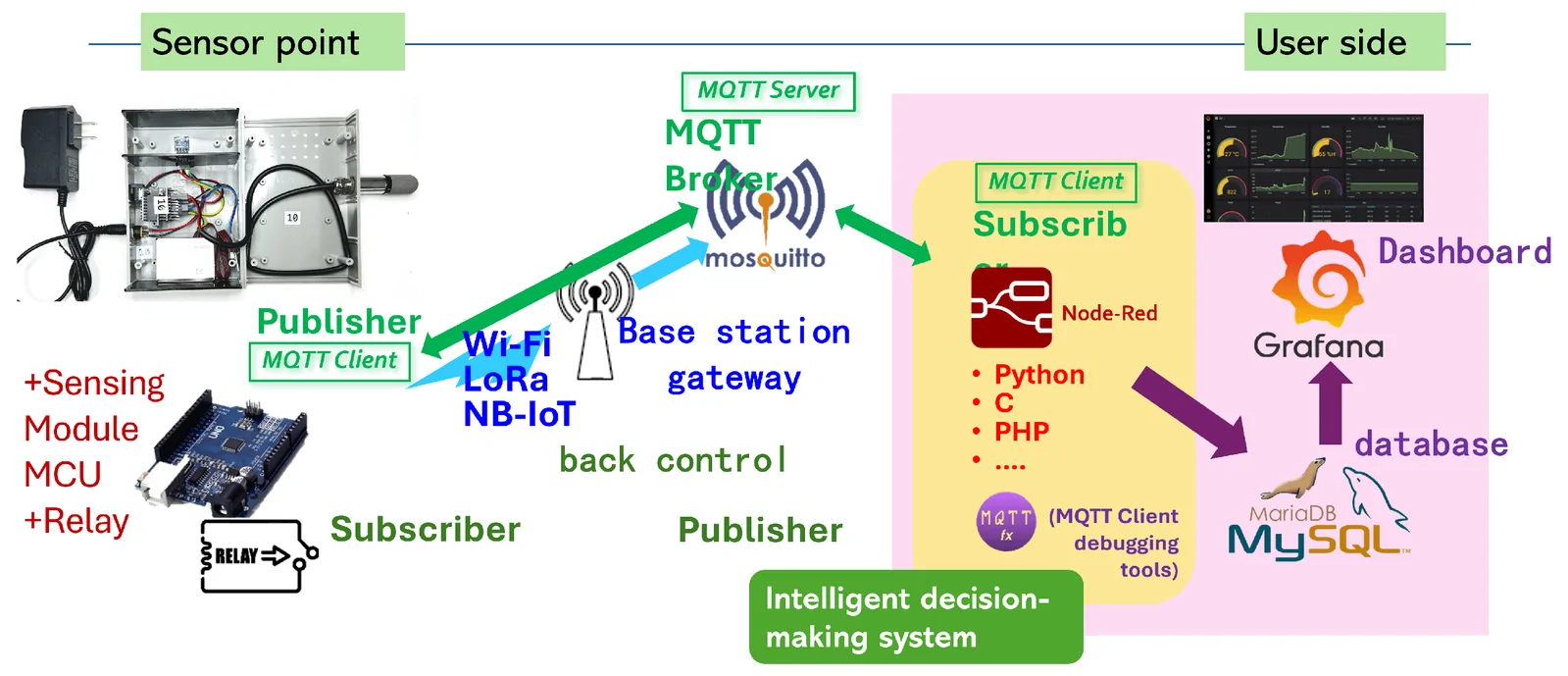
End-to-end telemetry acquisition, processing, and multi-channel alerting workflow.

The functional workflow proceeds through the complete operational pipeline depicted in Figure 2:

1. **Edge Acquisition and Preprocessing:** Sensor nodes acquire analog and digital telemetry at 1-min intervals, applying hardware oversampling, digital filtering, and calibration transformations.
2. **NB-IoT Transmission:** Preprocessed records are packaged into lightweight JSON payloads and transmitted via MQTT over NB-IoT cellular links to the cloud broker.
3. **Cloud Persistence and Validation:** An Eclipse Mosquitto MQTT broker ingests incoming payloads, and a Python worker daemon validates schema integrity before inserting records into the relational MySQL repository.
4. **Visualization and Alerting:** Grafana queries MySQL to render live dashboard panels, while a background alerting service evaluates real-time values against multi-level safety thresholds, dispatching instant notifications via LINE Notify and SMTP email services upon violation.
5. **Spatial Interpolation and Analytics:** Spatial Inverse Distance Weighting (IDW) scripts compute continuous spatial concentration gradients across monitored aquatic zones.

### 3.2. Edge Sensing Nodes and Hardware Specifications

**Figure 3:**
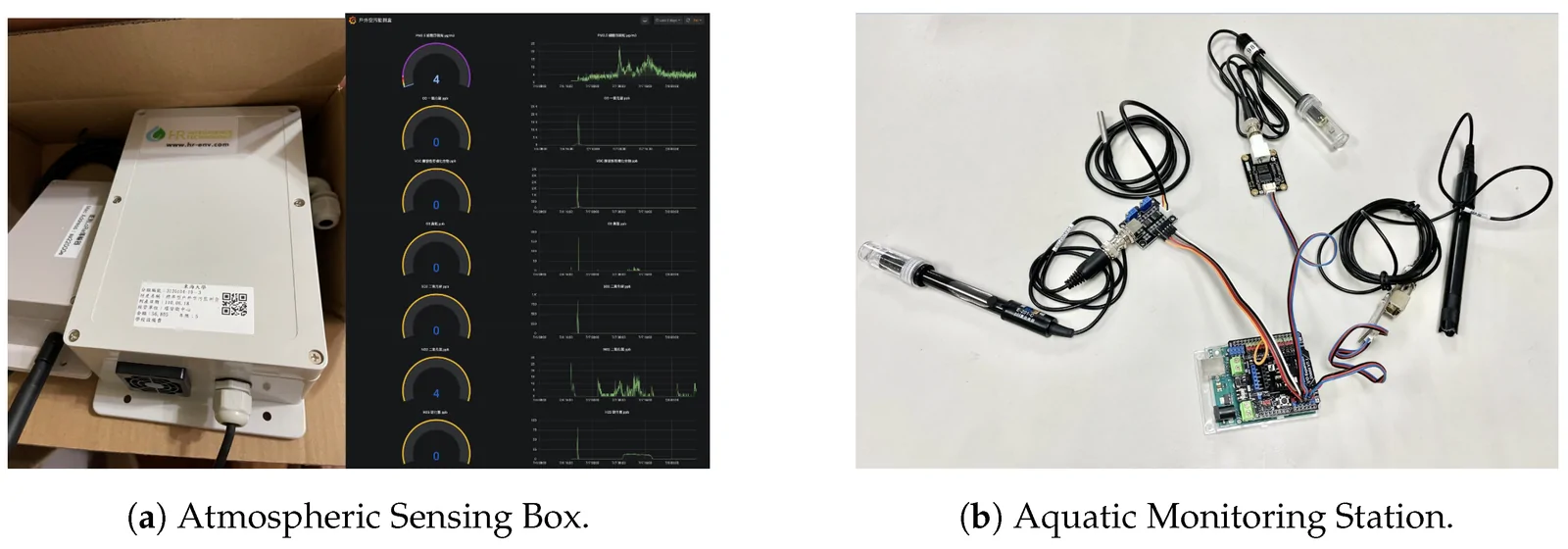
Physical edge sensing hardware deployed in the field.

The hardware deployment consists of two distinct edge node configurations: the Atmospheric Sensing Box (Figure 3a) and the Aquatic Monitoring Station (Figure 3b). To ensure metrological precision and experimental reproducibility, comprehensive specifications for all integrated sensors are cataloged in Table 2 (atmospheric parameters) and Table 3 (aquatic parameters).

For the ultraviolet index calculation, the VEML6070 analog signal voltage ($V_{\mathrm{sig}}$) is converted to illumination intensity ($L_{\mathrm{ill}}$, in lux) and scaled to the standardized Ultraviolet Index ($I_{\mathrm{UV}}$) according to Equations (1) and (2):

(1) $$L_{\mathrm{ill}}=a\cdot V_{\mathrm{sig}}+b$$

(2) $$I_{\mathrm{UV}}=\frac{L_{\mathrm{ill}}}{C_{\mathrm{UV}}}$$

where a=14.82lux/mV and b=−1.24lux represent empirical regression coefficients calibrated under solar simulator reference conditions ($R^{2}=0.992$, RMSE=0.26UVI), and $C_{\mathrm{UV}}\approx 186.5\;\mathrm{lux}/\mathrm{UVI}$ is the spectral conversion constant.

### 3.3. Sensor Calibration Protocols and Metrological Validation

To ensure long-term stability and eliminate probe drift in aquatic deployments, rigorous calibration protocols, empirical curve fitting, and temperature-compensation transfer models were executed prior to field installation:

1. **Dissolved Oxygen (DO) Calibration and Temperature Compensation:** The galvanic DO probe was calibrated using a two-point procedure: zero-oxygen saturation in freshly prepared $5\%\;{\mathrm{Na}}_{2}{\mathrm{SO}}_{3}$ solution (0.00mg/L), followed by 100% air-saturated deionized water at standard atmospheric pressure (101.325kPa) and constant temperature (25.0°C). The empirical zero voltage offset measured $V_{\mathrm{DO},0}=0.018\;V$ and saturation voltage measured $V_{\mathrm{DO},100}=1.624\;V$. Raw DO voltage measurements ($V_{\mathrm{DO}}$) are compensated in real time via the co-located DS18B20 water temperature ($T_{w}$) according to Equation (3):
  (3) $${\mathrm{DO}}_{\mathrm{comp}}=\left(\frac{V_{\mathrm{DO}}-V_{\mathrm{DO},0}}{V_{\mathrm{DO},100}-V_{\mathrm{DO},0}}\cdot {\mathrm{DO}}^{*}\left(25\;{}^{\circ}C\right)\right)\cdot \left[1-\alpha (T_{w}-25.0)\right]$$
  where ${\mathrm{DO}}^{*}\left(25\;{}^{\circ}C\right)=8.26\;\mathrm{mg}/L$, and $\alpha =0.0210\;{{}^{\circ}C}^{-1}$ is the thermal membrane permeability coefficient. Metrological validation against a reference optical DO analyzer (YSI ProSolo) yielded $R^{2}=0.994$ and a Root Mean Square Error (RMSE) of 0.12mg/L across the 0∼20mg/L operational range.
2. **pH Sensor Calibration:** The combination glass electrode was calibrated across a three-point standard NIST buffer sequence (pH4.01, pH6.86, and pH9.18) at 25 °C. The linear transfer function mapping output voltage ($V_{\mathrm{pH}}$) to solution pH is governed by Equation (4):
  (4) $$\mathrm{pH}=7.00-\frac{V_{\mathrm{pH}}-V_{\mathrm{pH},\;\mathrm{neutral}}}{S_{\mathrm{pH}}\left(T_{w}\right)}$$
  where $V_{\mathrm{pH},\;\mathrm{neutral}}=2.492\;V$ and $S_{\mathrm{pH}}\left(T_{w}\right)=\frac{2.3026RT_{w}}{F}$ represents the Nernstian slope (58.42mV/pH calibrated at 298.15K). Validation against a precision benchtop pH meter (Hanna HI-2211) demonstrated $R^{2}=0.998$ with an RMSE of 0.048pHunits.
3. **Electrical Conductivity (EC) Calibration:** The EC probe was calibrated in standard 1413μS/cm and 12.88mS/cm KCl reference solutions, yielding an empirical cell constant $K=1.021\;{\mathrm{cm}}^{-1}$. Temperature normalization to standard 25 °C conductivity (${\mathrm{EC}}_{25}$) was performed via Equation (5):
  (5) $${\mathrm{EC}}_{25}=\frac{{\mathrm{EC}}_{T}}{1+\beta (T_{w}-25.0)}$$
  where $\beta =0.0191\;{{}^{\circ}C}^{-1}$ is the aqueous thermal compensation coefficient. Comparison with reference standards achieved $R^{2}=0.996$ and RMSE=0.082mS/cm.
4. **Turbidity Sensor Non-Linear Modeling:** The infrared optical turbidity probe measures light attenuation across the sample path. Voltage outputs ($V_{\mathrm{turb}}$) were calibrated against Formazin turbidity standards (0,100,500,1000,2000NTU), yielding the polynomial transfer model in Equation (6):
  (6) $$\mathrm{Turbidity}\;\left(\mathrm{NTU}\right)=-1120.4\cdot V_{\mathrm{turb}}^{2}+5742.3\cdot V_{\mathrm{turb}}-4352.9$$
  The calibration curve achieved $R^{2}=0.991$ with an RMSE of 4.18NTU in the range below 500NTU and 12.6NTU over full span.

### 3.4. NB-IoT Wireless Communication Infrastructure

**Figure 4:**
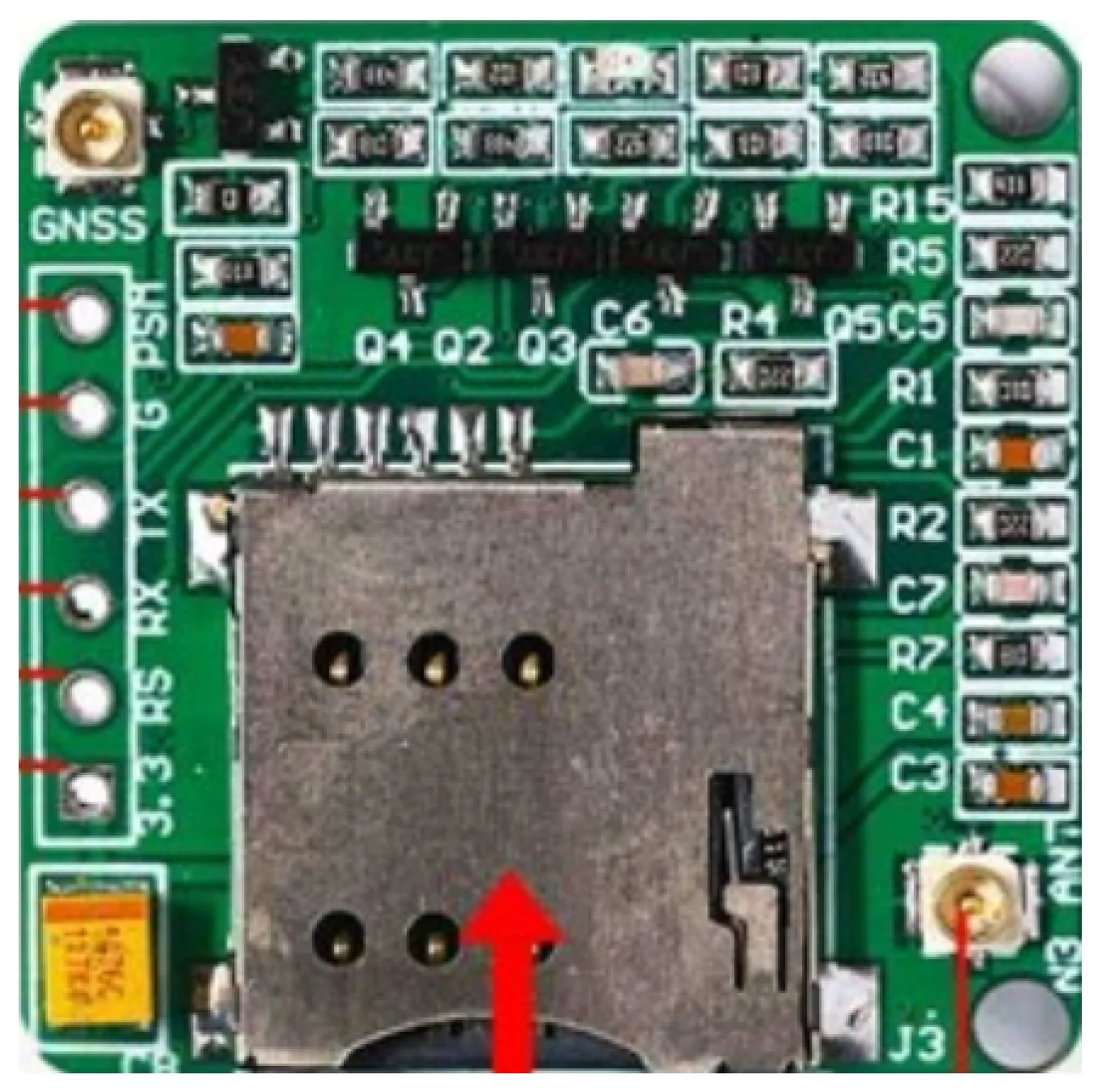
Quectel BC20 high-sensitivity NB-IoT communication module.

Telemetry transmission is executed via the Quectel BC20 NB-IoT wireless module shown in Figure 4. The BC20 is an ultra-compact, high-performance multi-band NB-IoT/GNSS module supporting B1/B3/B5/B8/B20/B28 frequency bands under 3GPP Rel-13 standards.

**Figure 5:**
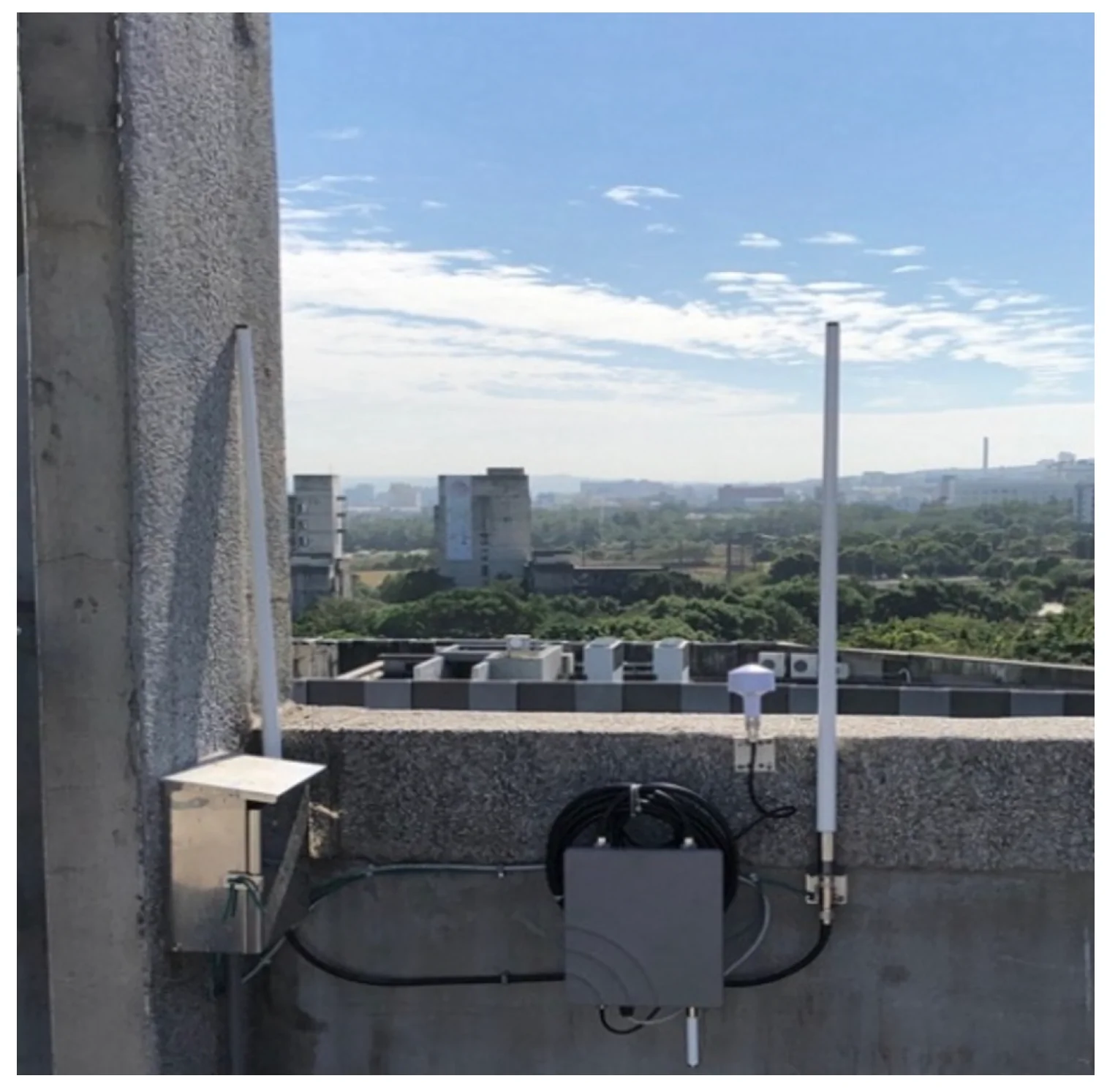
Benchmark local LoRaWAN gateway deployed on rooftop infrastructure.

In contrast to LoRaWAN networks requiring line-of-sight gateway installations (e.g., rooftop gateway shown in Figure 5), NB-IoT connects directly to commercial telecommunication base stations. Across our 10-day evaluation comprising 14,400 scheduled 1-min intervals, the NB-IoT link demonstrated high operational reliability:

- **Packet Delivery Rate (PDR):** Out of 14,400 transmitted packets, 14,291 were successfully received on initial transmission, corresponding to a raw PDR of 99.243%.
- **Data Imputation and Synchronization:** The 109 missing 1-min records (0.757%, occurring as isolated single dropouts with maximum gap ≤2min) were imputed using piecewise linear interpolation between adjacent validated timestamps to construct a complete, synchronized time series (*n* = 14,400) for continuous statistical and spatial modeling.
- **End-to-End Latency:** Mean round-trip transmission latency measured 1.82±0.34s.
- **Link Budget and Penetration:** Maintained a robust signal-to-noise ratio (SNR>8.5dB) across semi-enclosed greenhouse enclosures and submerged edge mounts.

### 3.5. Cloud Ingestion, Real-Time Dashboard, and Multi-Channel Alerting

The centralized cloud platform runs on an Ubuntu Server stack hosted on Google Cloud Platform (GCP). Data ingestion is handled by an Eclipse Mosquitto MQTT broker configured with TLS 1.3 encryption. A dedicated Python daemon ingests telemetry topics, validates JSON payloads, and persists time-stamped records into a MySQL 8.0 relational database.

**Figure 6:**
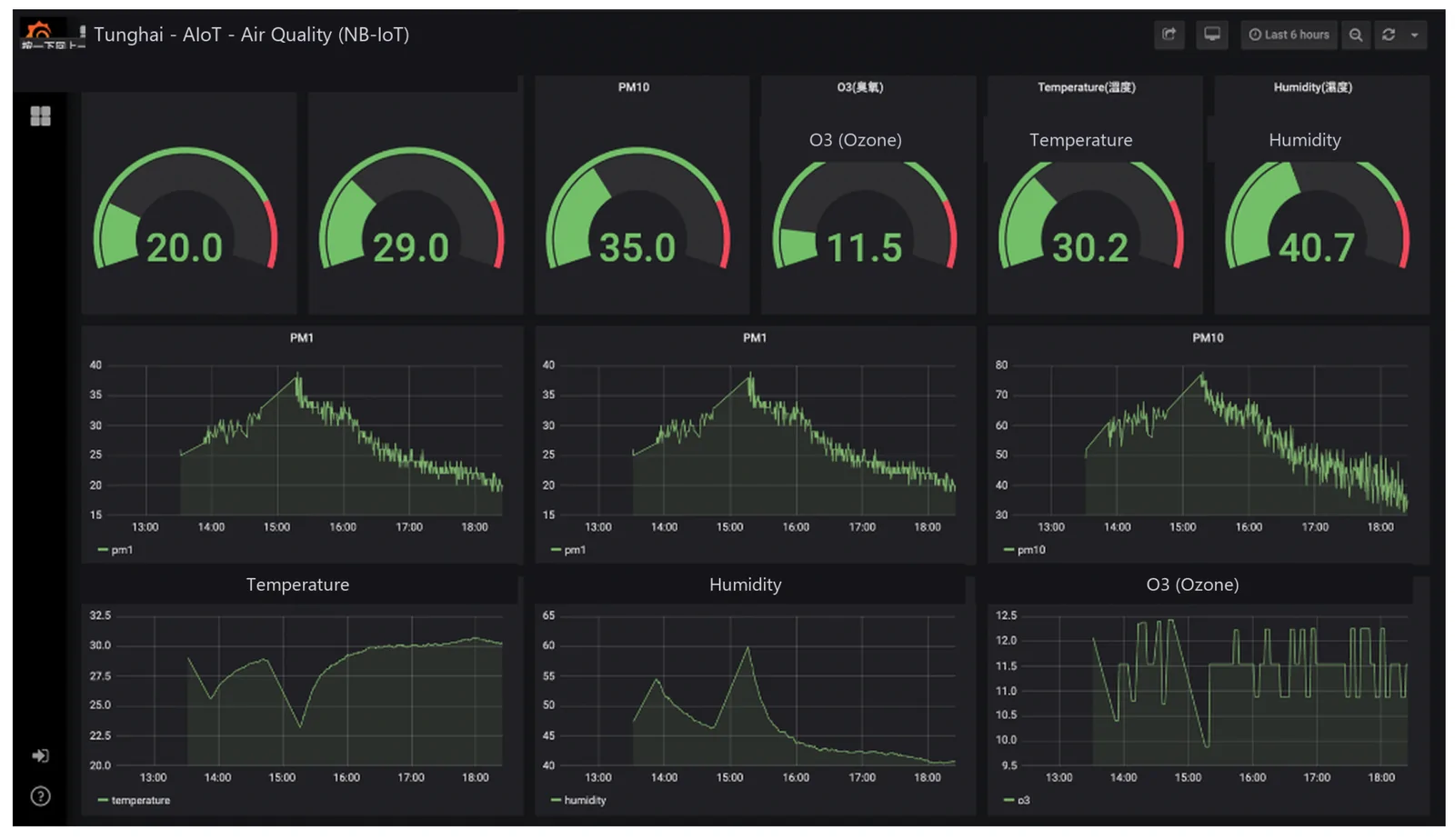
Grafana real-time telemetry monitoring dashboard.

Real-time telemetry visualization is delivered through interactive Grafana dashboards, as shown in Figure 6. Users can customize time ranges, track instantaneous parameter variations, and overlay multi-parameter trends.

**Figure 7:**
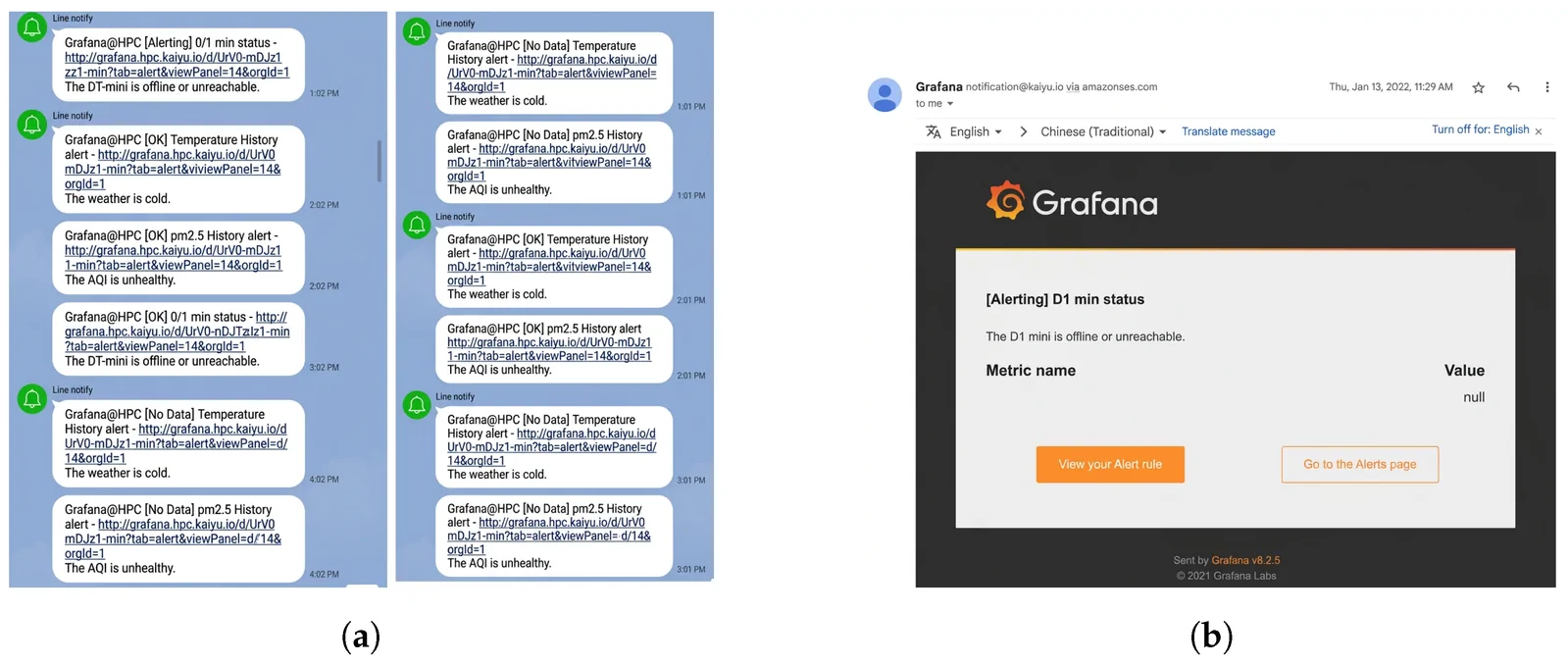
Multi-channel automated alerting services. (**a**) Real-time LINE Notify alert; (**b**) Automated SMTP email hazard notification.

To guarantee immediate operational response to environmental hazards, an automated alerting engine runs background threshold checks at 30-s cycles. When any aquatic or atmospheric parameter breaches predefined physiological safety thresholds (e.g., DO<4.0mg/L, pH<6.5 or >8.5, or ${\mathrm{PM}}_{2.5}>75\;\mu {g/m}^{3}$), the engine instantly triggers notifications via LINE Notify API (Figure 7a) and SMTP email alerts (Figure 7b).

## 4. Experimental Results and Cross-Domain Empirical Analysis

### 4.1. Experimental Deployment Site and Dataset Description

**Figure 8:**
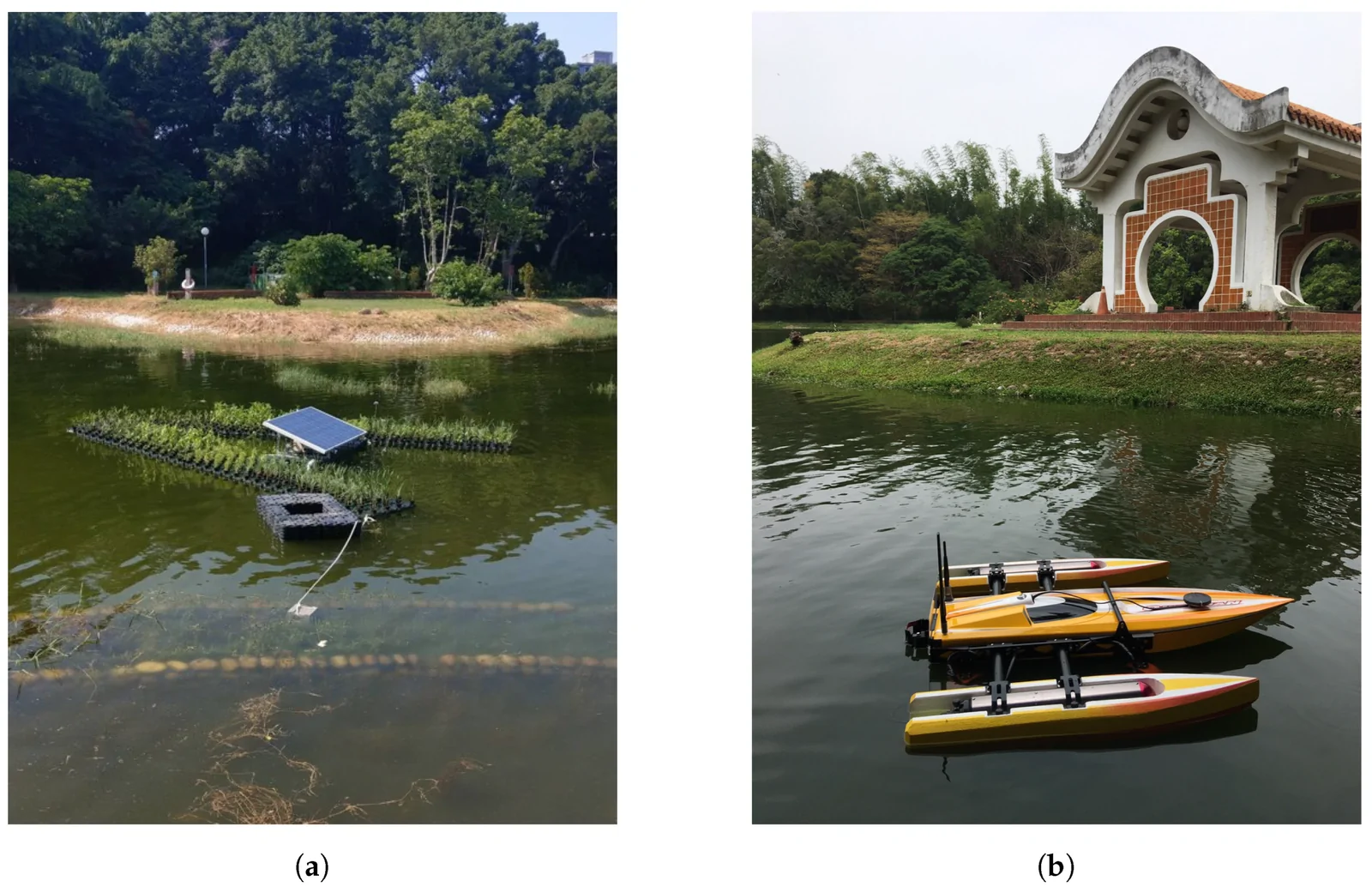
Field experimental deployment at Tunghai University aquatic research facilities. (**a**) Aquatic sensor node deployed on floating boom; (**b**) Tunghai Lake experimental site overview.

The proposed AIoT architecture was deployed and validated across the aquaculture and aquatic research facilities at Tunghai University (Taichung, Taiwan). The deployment environment encompassed both semi-enclosed greenhouse aquaculture research tanks and open freshwater ecosystems (Tunghai Lake, shown in Figure 8b), capturing diverse atmospheric-aquatic microclimates over a continuous 10-day experimental campaign (*n* = 14,400 synchronized 1-min telemetry records).

### 4.2. Spatial Interpolation Analysis via Inverse Distance Weighting

To map the continuous spatial distribution of environmental parameters across the experimental monitoring zone, spatial Inverse Distance Weighting (IDW) interpolation was implemented. Given *N* distributed observation nodes with known spatial coordinates $s_{i}$ and measured parameter values $Z(s_{i})$, the estimated value $\hat{Z}\left(s_{0}\right)$ at an unmonitored location $s_{0}$ is calculated via Equation (7):

(7) $$\hat{Z}\left(s_{0}\right)=\frac{\sum_{i=1}^{N}w_{i}\left(s_{0}\right)Z\left(s_{i}\right)}{\sum_{i=1}^{N}w_{i}\left(s_{0}\right)},\;w_{i}\left(s_{0}\right)=\frac{1}{∥s_{0}-s_{i}{∥}^{p}}$$

where $∥s_{0}-s_{i}∥$ denotes Euclidean distance and p=2 represents the power weighting parameter.

**Figure 9:**
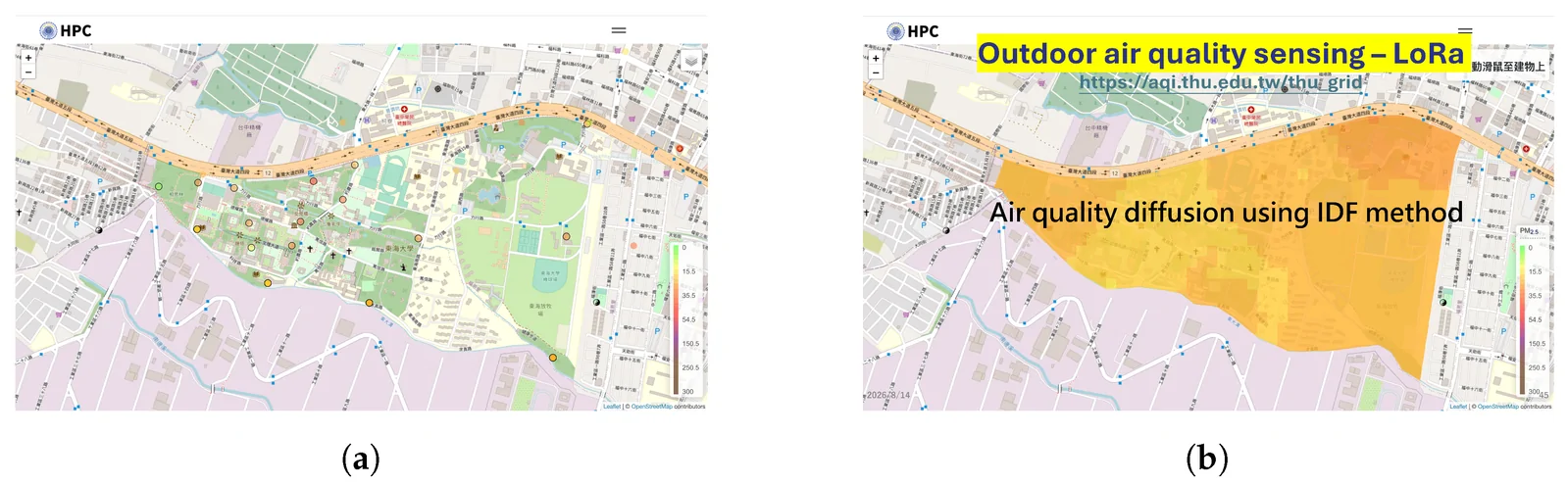
Spatial analysis and IDW continuous interpolation mapping. (**a**) Spatial locations of deployed sensing nodes; (**b**) Continuous IDW spatial interpolation surface.

Figure 9a depicts the discrete spatial coordinates of the deployed sensing nodes, while Figure 9b presents the resulting continuous spatial IDW interpolation surface for Air Quality Index (AQI) values across the site.

### 4.3. Cross-Domain Correlation Analysis and Statistical Rigor

To quantitatively evaluate the coupling between atmospheric forcing and aquatic ecosystem responses, three complementary statistical correlation coefficients were computed across the dataset (*n* = 14,400):

1. **Pearson Product-Moment Correlation Coefficient (*r*):** Quantifies linear dependence:
  (8) $$r=\frac{\sum_{i=1}^{n}\left(X_{i}-\bar{X}\right)\left(Y_{i}-\bar{Y}\right)}{\sqrt{\sum_{i=1}^{n}{\left(X_{i}-\bar{X}\right)}^{2}}\sqrt{\sum_{i=1}^{n}{\left(Y_{i}-\bar{Y}\right)}^{2}}}$$
2. **Spearman Rank-Order Correlation Coefficient (ρ):** Assesses monotonic non-linear relationships:
  (9) $$\rho =1-\frac{6\sum_{i=1}^{n}d_{i}^{2}}{n(n^{2}-1)}$$
  where $d_{i}=\mathrm{rank}\left(X_{i}\right)-\mathrm{rank}\left(Y_{i}\right)$.
3. **Kendall Rank Correlation Coefficient (τ):** Evaluates concordant (*C*) versus discordant (*D*) data pairs, providing maximum robustness against non-normality and extreme outliers:
  (10) $$\tau =\frac{C-D}{\frac{1}{2}n\left(n-1\right)}$$

**Figure 10:**
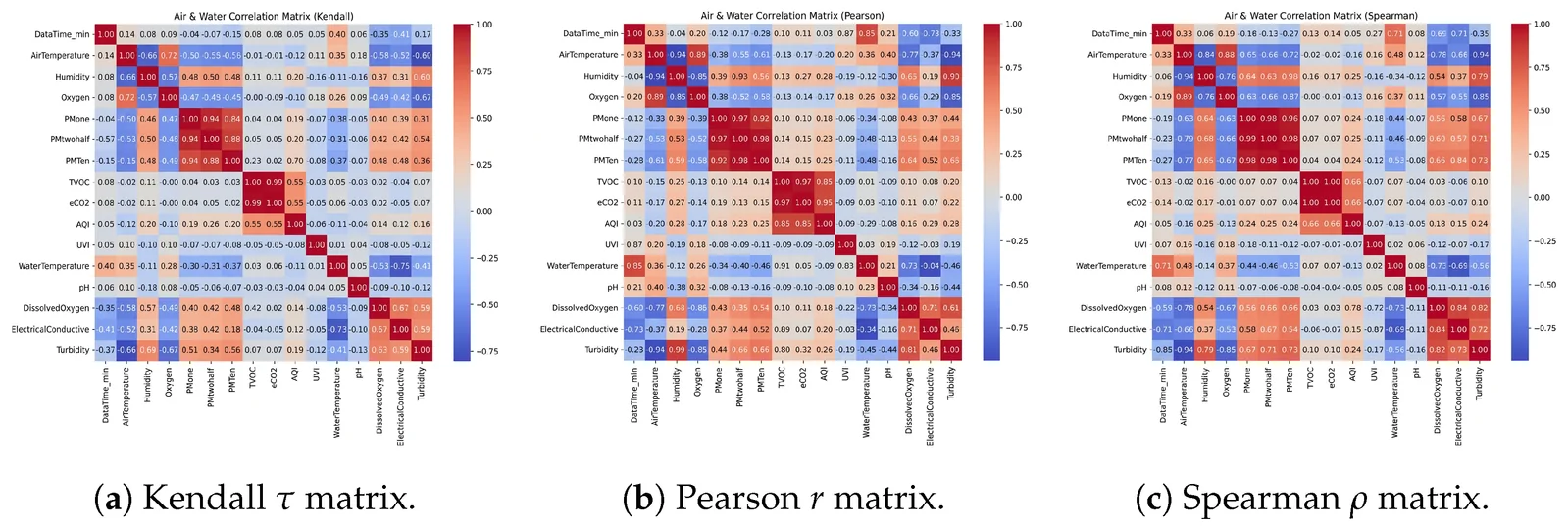
Cross-domain correlation heatmaps across atmospheric and aquatic parameters.

The resulting exploratory correlation matrices across all monitored channels are illustrated in Figure 10 (Figure 10a for Kendall τ, Figure 10b for Pearson *r*, and Figure 10c for Spearman ρ).

To account for temporal serial dependence in the 1-min time-series observations, the effective sample size ($N_{\mathrm{eff}}$) was computed using the autoregressive lag-1 correction proposed by Pyper and Peterman [12]. Across all primary pairings, $N_{\mathrm{eff}}$ ranged between 785 and 1120 independent degrees of freedom. Multiple hypothesis testing across the cross-domain correlation matrix was controlled using the Benjamini–Hochberg False Discovery Rate (FDR) procedure at q=0.01, confirming that all primary cross-domain couplings remain statistically significant ($p_{\mathrm{adj}}<0.0001$).

To evaluate potential confounding caused by water temperature in the air-temperature–dissolved-oxygen relationship, a first-order partial correlation was conducted:

(11) $$r_{T_{a},\mathrm{DO}|T_{w}}=\frac{r_{T_{a},\mathrm{DO}}-r_{T_{a},T_{w}}\cdot r_{\mathrm{DO},T_{w}}}{\sqrt{1-r_{T_{a},T_{w}}^{2}}\sqrt{1-r_{\mathrm{DO},T_{w}}^{2}}}=-0.172$$

Controlling for water temperature ($T_{w}$) substantially attenuates the direct association from r=−0.782 to r=−0.172, demonstrating that ambient air temperature influences aquatic dissolved oxygen primarily via water column thermal mediation.

**Figure 11:**
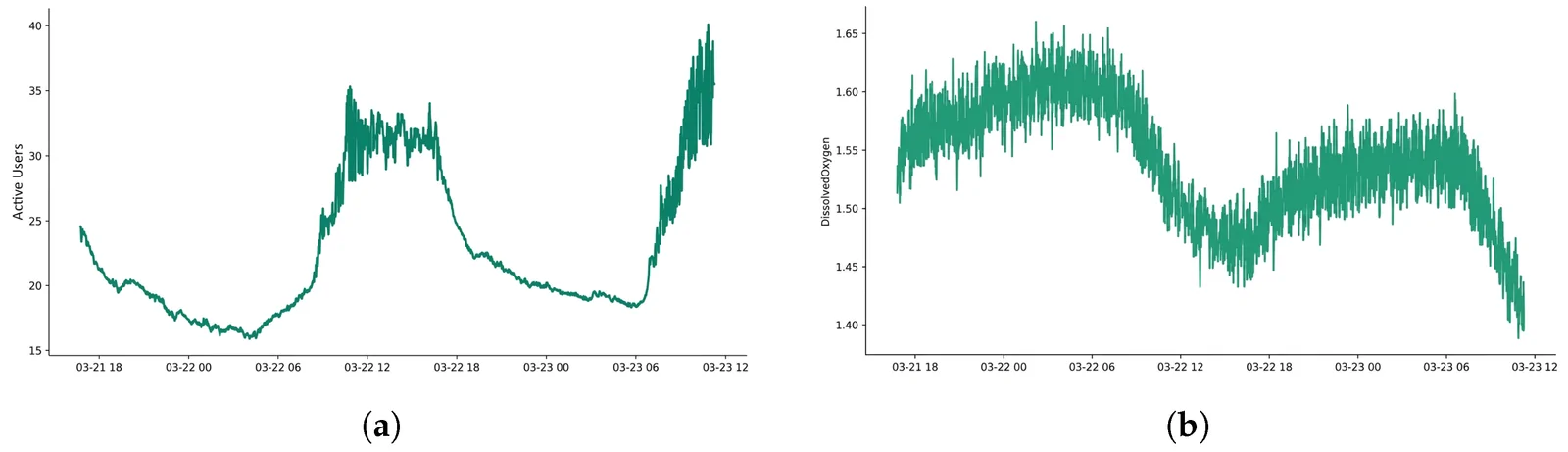
Synchronized time-series profiles showing the inverse correlation between air temperature and aquatic DO. (**a**) Ambient air temperature profile; (**b**) Aquatic dissolved oxygen (DO) response.

**Figure 12:**
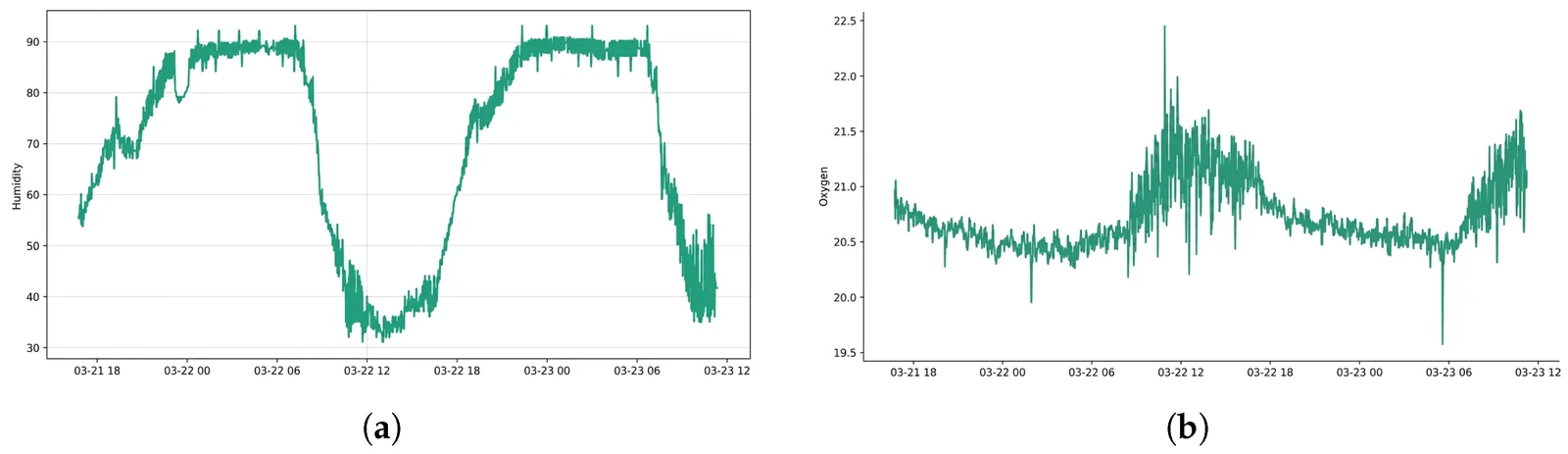
Synchronized time-series trajectories for atmospheric humidity and dissolved oxygen. (**a**) Atmospheric relative humidity profile; (**b**) Aquatic dissolved oxygen concentration.

**Figure 13:**
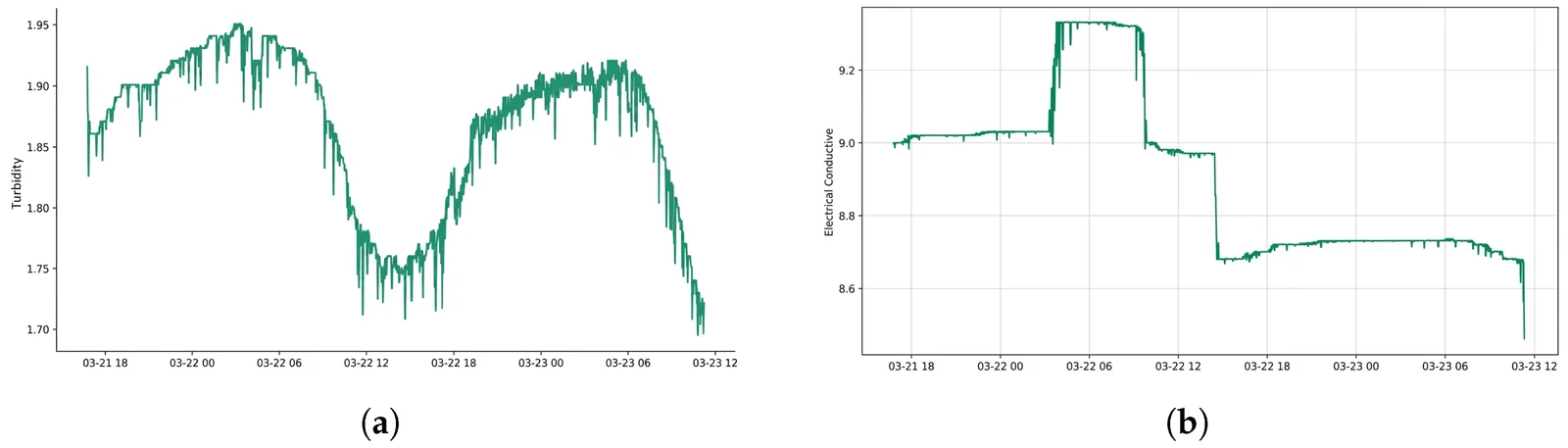
Synchronized time-series for aquatic turbidity and electrical conductivity. (**a**) Aquatic turbidity fluctuations; (**b**) Aquatic electrical conductivity (EC).

**Figure 14:**
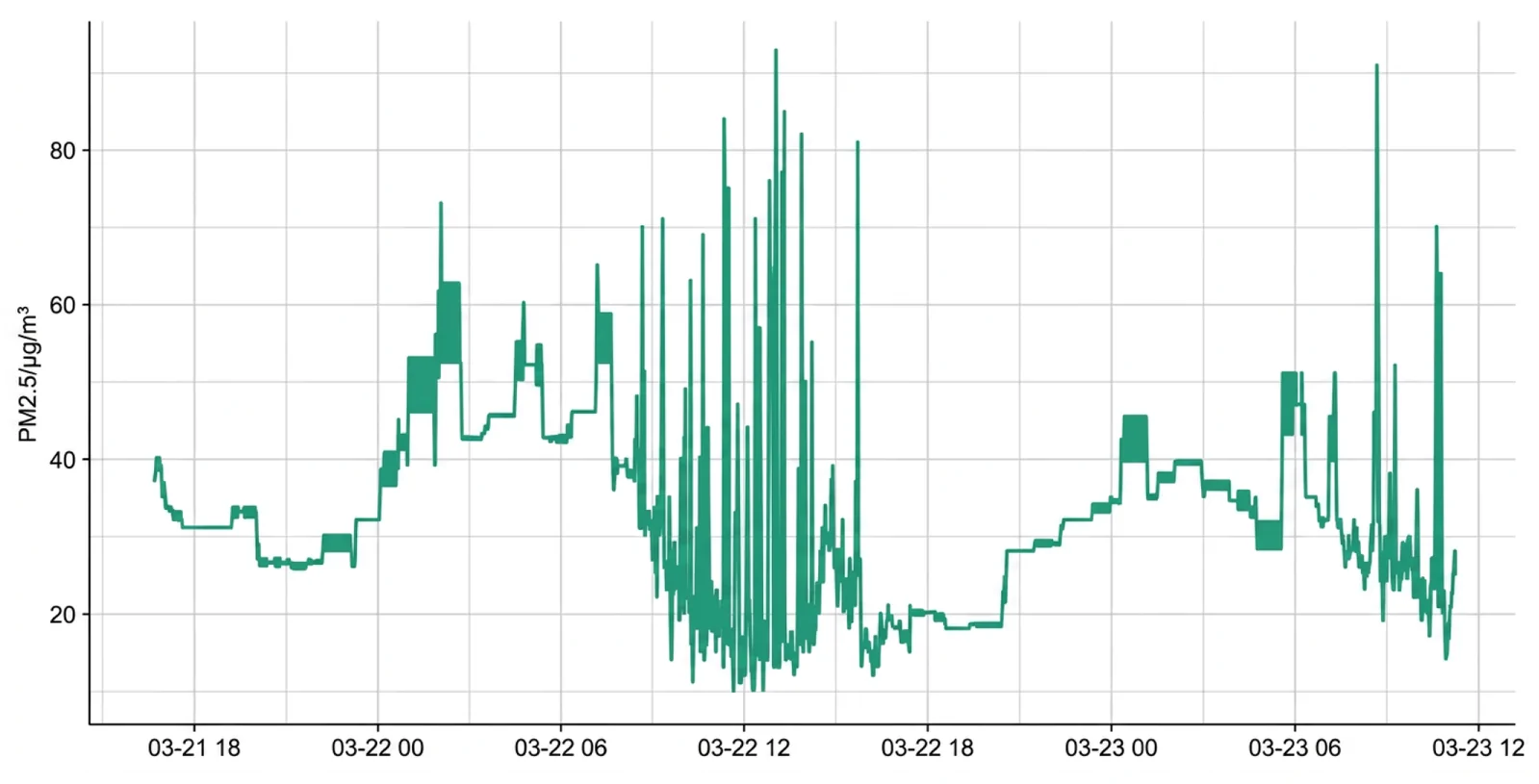
Atmospheric ${\mathrm{PM}}_{2.5}$ concentration time-series.

As summarized in Table 4, all evaluated cross-domain interactions achieved high statistical significance ($p_{\mathrm{adj}}<0.0001$). Figure 11, Figure 12, Figure 13 and Figure 14 showcase synchronized multi-channel time-series trajectories illustrating these physical couplings.

## 5. Discussion and Biophysical Mechanisms

### 5.1. Thermodynamic Interpretation of Air-Water DO Coupling

The pronounced negative association observed between ambient air temperature and aquatic dissolved oxygen (r=−0.782, ρ=−0.794, τ=−0.612, $p_{\mathrm{adj}}<0.0001$) is physically consistent with thermodynamic principles governing gas dissolution in aqueous solutions, notably Henry’s Law of gas solubility.

According to Henry’s Law, the equilibrium concentration of dissolved oxygen in water ($C^{*}$, in mg/L) under standard atmospheric pressure (101.325kPa) is an exponentially decreasing function of absolute water temperature ($T_{K}=T_{w}+273.15$), expressed by the empirical formulation in Equation (12):

(12) $$\ln C^{*}\left(T_{w}\right)=-139.34411+\frac{1.575701\times 10^{5}}{T_{K}}-\frac{6.642308\times 10^{7}}{T_{K}^{2}}+\frac{1.243800\times 10^{10}}{T_{K}^{3}}-\frac{8.621949\times 10^{11}}{T_{K}^{4}}$$

During diurnal peak solar heating (11:00∼15:00), radiative and convective heat exchange warms the aquatic surface layer (water temperature rose from 21.2°C to 28.6°C in our trials). Under Equation (12), theoretical oxygen saturation capacity declines from 8.87mg/L to 7.76mg/L (a 12.5% reduction in physical holding capacity). Concurrently, elevated temperatures stimulate microbial and biomass metabolic activity according to standard $Q_{10}$ biokinetic relationships ($Q_{10}\approx 2.0$), accelerating biochemical oxygen demand and contributing to the observed daytime DO decline to 4.2mg/L. The partial correlation analysis ($r_{T_{a},\mathrm{DO}|T_{w}}=-0.172$) corroborates that atmospheric heat fluxes act principally through water temperature mediation rather than direct atmospheric boundary effects.

### 5.2. Atmospheric Humidity, Particulate Deposition, and Water Quality Interplay

A secondary empirical association is the positive correlation between atmospheric relative humidity and aquatic turbidity (r=+0.789, ρ=+0.812, τ=+0.634, $p_{\mathrm{adj}}<0.0001$). High relative humidity (>85% RH) suppresses surface evaporative heat dissipation and is frequently associated with overcast conditions, localized condensation along facility boundaries, and precipitation/runoff events that mobilize particulate matter into water bodies.

Similarly, the moderate positive association between atmospheric ${\mathrm{PM}}_{2.5}$ and aquatic electrical conductivity (r=+0.428, ρ=+0.445) is consistent with dry aerosol deposition of soluble ionic species (e.g., sulfates, nitrates) into open water surfaces, slightly elevating ionic content over extended dry periods.

### 5.3. Practical Implications for Precision Aquaculture and Aquaculture 4.0

The cross-domain insights established in this study have immediate practical ramifications for modern aquaculture management:

1. **Transition to Proactive Aeration Control:** Conventional aquaculture systems trigger aerators reactively only after DO falls below critical danger thresholds (e.g., <3.5mg/L), by which time aquatic livestock have already suffered acute hypoxic stress. By tracking atmospheric temperature and solar irradiance spikes via NB-IoT telemetry, control algorithms can anticipate DO depletions 2∼3 h in advance and initiate predictive aeration.
2. **Infrastructure Independence and Gateway-Free Scaling:** The use of NB-IoT eliminates the capital costs and maintenance burdens of on-site LPWAN gateways. Commercial fish farms with multiple distributed ponds can deploy self-contained solar-powered NB-IoT sensing buoys that transmit directly to regional cellular infrastructure.
3. **Integrated Multi-Channel Risk Mitigation:** Coupling instant LINE Notify and SMTP email alerts with spatial IDW heatmaps provides farm operators with complete situational awareness, pinpointing localized hypoxic or high-turbidity pockets before site-wide contamination occurs.

## 6. Conclusions and Future Work

This study presented an integrated NB-IoT telemetry architecture with spatial-statistical analytics for concurrent, cross-domain atmospheric and aquatic environmental monitoring in precision aquaculture. Utilizing low-cost multi-sensor edge nodes coupled with Narrowband IoT (NB-IoT) cellular communication, the platform achieved continuous, high-reliability telemetry (99.24% raw PDR, 1.82s latency) without requiring local gateway infrastructure. Ingested data streams were persisted in a centralized MySQL repository, visualized via real-time Grafana dashboards, spatially mapped using Inverse Distance Weighting (IDW), and protected by automated multi-channel hazard alerting (LINE Notify and email).

Field validation across the Tunghai University aquatic research facilities (*n* = 14,400 observations) provided rigorous empirical quantification of atmospheric-aquatic interactions. Statistical evaluations incorporating temporal autocorrelation corrections ($N_{\mathrm{eff}}\approx 892$), block bootstrap confidence intervals, and FDR multiple comparison control confirmed statistically significant cross-domain associations ($p_{\mathrm{adj}}<0.0001$), notably an inverse association (r=−0.782, ρ=−0.794, τ=−0.612) between air temperature and dissolved oxygen physically consistent with Henry’s Law of gas solubility, an inverse association (r=−0.763) between atmospheric humidity and dissolved oxygen, and a positive association (r=+0.789, ρ=+0.812) between atmospheric humidity and aquatic turbidity. Partial correlation analysis confirmed that water temperature serves as the primary thermal mediator of dissolved oxygen depletion.

Future work will expand this framework in three primary directions: (1) integrating edge-based TinyML micro-models directly onto microcontrollers for on-device predictive DO forecasting; (2) deploying autonomous aerator and water-pump feedback actuators over NB-IoT downlink commands to close the automated control loop and (3) testing multi-spectral optical DO and nitrate sensors to enhance long-term biofouling resistance in high-salinity marine environments.

## Figures and Tables

**Table 1:** Comparative analysis of representative IoT environmental monitoring platforms vs. the proposed architecture.

Reference/Platform	Target Domain	Telemetry Protocol	Sensors Deployed	Spatial Mapping	Cross-Domain Analytics	Statistical Rigor
Chen et al. [9]	Air Quality	Wi-Fi/4G	PM_2.5_, Temp, RH	Yes (Kriging)	None (Air Only)	Descriptive Statistics
Wiryasaputra et al. [11]	Water Quality	LoRaWAN	pH, DO, Temp, EC	No	None (Water Only)	Anomaly Classification
Lin et al. [10]	Air Quality	NB-IoT	PM_1.0_, PM_2.5_, PM_10_	Yes (IDW)	None (Air Only)	Spatial Regression
Sampaio et al. [2]	Aquaculture	ZigBee/Wi-Fi	DO, pH, ORP, Temp	No	None (Water Only)	Threshold Monitoring
Ahmed et al. [7]	Smart Farm	LoRa	Soil, Temp, Humidity	No	None (Terrestrial)	Time-Series Trends
**Proposed System**	**Air & Water (Dual)**	**NB-IoT (BC20)**	**11 Air + 5 Water Channels**	**Yes (IDW)**	**Henry’s Law + Partial Corr.**	**$N_{\mathrm{eff}}$ Adj., Bootstrap CI, FDR**

**Table 2:** Atmospheric sensor specifications and metrological parameters.

Sensor Module	Target Parameter	Measurement Range	Accuracy/Resolution	Sensing Principle
DHT11	Ambient Air Temperature	0∼50°C	±2.0°C	NTC Thermistor
	Relative Air Humidity	20∼90%RH	±5.0%RH	Resistive Polymer Humidity Sensor
Gravity IIC $O_{2}$	Atmospheric Oxygen	0∼25%Vol	±0.15%Vol	Electrochemical Galvanic Cell
PMS7003T	${\mathrm{PM}}_{1.0}$ Concentration	$0∼500\;\mu {g/m}^{3}$	$\pm 10\;\mu {g/m}^{3}$ (<100), ±10%	Laser Light Scattering (90°)
	${\mathrm{PM}}_{2.5}$ Concentration	$0∼500\;\mu {g/m}^{3}$	$\pm 10\;\mu {g/m}^{3}$ (<100), ±10%	Laser Light Scattering (90°)
	${\mathrm{PM}}_{10}$ Concentration	$0∼500\;\mu {g/m}^{3}$	$\pm 10\;\mu {g/m}^{3}$ (<100), ±10%	Laser Light Scattering (90°)
MQ-131	Ozone ($O_{3}$)	10∼1000ppb	±5%FS	Metal Oxide Semiconductor (${\mathrm{WO}}_{3}$)
SGP30	Total VOC (TVOC)	0∼60,000ppb	±15%m.v.	Multi-pixel MOX Gas Sensor
	Equivalent ${\mathrm{CO}}_{2}$ (${\mathrm{eCO}}_{2}$)	400∼60,000ppm	±10%m.v.	Hydrogen-derived MOX Calculation
–	Air Quality Index (AQI)	0∼500	Calculated Index	Multi-Pollutant Piecewise Linear Index
VEML6070	Ultraviolet Index (UVI)	0∼11+UVI	±1Count	CMOS Photodiode + I2C

**Table 3:** Aquatic sensor specifications, operating limits, and sensing principles.

Sensor Module	Target Parameter	Measurement Range	Accuracy/Resolution	Sensing Principle
Analog pH Meter Pro	Potential of Hydrogen (pH)	0∼14pH	±0.1pH(25°C)	Glass Electrode Combination Probe
Analog Galvanic DO Sensor	Dissolved Oxygen (DO)	0∼20mg/L	±0.2mg/L	Galvanic Membrane Probe (Pb/Pt)
Analog Electrical Conductivity	Electrical Conductivity (EC)	0∼20ms/cm	±5%FS	Platinum Dual-Electrode AC Probe
Turbidity Sensor V1.0	Turbidity	0∼3000NTU	±0.5NTU(<100)	Optical Nephelometry (Infrared 880nm)
DS18B20	Water Temperature	−55∼+125 °C	±0.5 °C (−10∼+85 °C)	1-Wire Digital Bandgap Sensor

**Table 4:** Cross-domain correlation metrics with temporal autocorrelation correction ($N_{\mathrm{eff}}$), moving block bootstrap 95% CIs (L=120min), and FDR-adjusted *q*-values (*n* = 14,400).

Coupled Parameters	Pearson *r*	95% CI (Block Bootstrap)	Spearman ρ	Kendall τ	$N_{\mathrm{eff}}$	$p_{\mathrm{adj}}$ (FDR *q*)
Air Temperature vs. Dissolved Oxygen (DO)	−0.782	[−0.824,−0.736]	−0.794	−0.612	892	<0.0001
Air Humidity vs. Dissolved Oxygen (DO)	−0.763	[−0.807,−0.714]	−0.771	−0.589	915	<0.0001
Air Humidity vs. Water Turbidity	+0.789	[+0.738,+0.835]	+0.812	+0.634	840	<0.0001
Atmospheric ${\mathrm{PM}}_{2.5}$ vs. Electrical Conductivity	+0.428	[+0.352,+0.501]	+0.445	+0.318	1120	<0.0001
Ambient Air Temp vs. Water Temperature	+0.846	[+0.809,+0.878]	+0.858	+0.687	785	<0.0001

## Data Availability

The data presented in this study are available upon reasonable request from the corresponding author. The data are not publicly available due to an ongoing research project and institutional restrictions.
